# Editorial: Global excellence in health policy and management: Africa

**DOI:** 10.3389/frhs.2025.1632681

**Published:** 2025-11-06

**Authors:** Godfrey Martin Mubyazi, Suzanne N. Kiwanuka

**Affiliations:** 1 National Institute for Medical Research (NIMR), Dar es Salaam, Tanzania; 2 Department of Health Policy Planning and Management, Makerere University, Kampala, Uganda

**Keywords:** global excellence, health policy and organization, Africa, sustainable health care, global collaboration

The standard English dictionaries define the word “excellence” as “an extraordinary quality” or “an extremely good condition.” However, this does not mean perfection. Excellence is assumed to be attainable with effort, skill, and strategy, but perfection is often assumed to be an unrealistic standard (1). Achieving excellence is a common pursuit in many life-related disciplines including that of policymaking and management. An “excellent policy” provides prospects for meeting the needs of the population or the community in question to a large degree, if not fully, within the admissible interpretational and practical limits or challenges (2–5). Great public policies are presumed to be coherent by design, comprehensive by content and coverage, while being consistent both within and between their field-based applications (6).

This Journal's research topic, titled “*Global Excellency In Health Policy and Management*: *Africa*,” attracted a number of manuscripts from the authors seeking to publish their work. In general, each team of authors underscored the urgency of strengthening national health policies and management systems based on reliable systematic evidence. The authors also emphasized that policies and practices need to be reviewed periodically in order to cope with contemporary needs and to enhance their acceptability, feasibility, sustainability, and attainment of desired outcomes.

The articles presented have addressed health policy and management issues in both broad and specific terms. Those by Isangula et al., Zenebe et al., and Okondo et al. focus on maternal-health service (MHS) policies and programs, while Yevoo et al. and Chekol et al. focus on quality of healthcare (QoHC) dimensions. Keleb et al., Okondo et al., and Mkumbo et al. underscore the factors hindering the ability of frontline healthcare workers (HCWs) to comply with the existing policy guidelines for delivering standard healthcare. Chaker Masmoudi et al. emphasize the need for existing or newly created government policies to be reasonably thought-out, in order to adequately support initiatives aimed at achieving an effective response to the challenges communities face in periods of emergency, the recent outbreak of COVID-19 being a recent example.

Reflecting on the findings from a study undertaken in several districts in rural Tanzania, Isangula et al. highlight the need for policies aimed at improving maternal and child health (MCH) to be clear and viable in order to facilitate their interpretation and uniform translation into action. They opine that reliable research evidence is crucial to inform such policies, although clear policy statements are not enough unless there are corresponding guidelines for their implementation. The authors also underscore the value of good relationships between nurses and their clients, which contribute to the realization of more positive service delivery outcomes. The same authors recommend the use of field-tested interventions to generate the evidence to inform the respective policies.

Zenebe et al. present a protocol for field-testing the feasibility and effectiveness of data-informed platforms for health, reasoning that this could contribute to guiding policy and management decisions, ultimately improving the delivery processes and outcomes of maternal health services (MHSs). The authors plan to test this proposition across several districts in Ethiopia. In their view, such an approach has the potential to minimize chronic health data/information distortions or wastage and therefore lead to increased MHS delivery improvements, service uptake, and MHS outcomes.

Okondo et al. present findings from a study conducted across five hospitals in Kenya to assess the compliance of HCWs with the standard person-centered care in practice. They report that shortages of key structural and process elements of the QoHC were a hindrance to HCWs' ability to comply with the recommended standards. These included hurdles caregivers faced when seeking care for their children; confusing user-fee payment modalities; the lack of feedback to caregivers about the diagnostics performed, treatment given, and the progress of the child's health. The authors believe that policy and program authorities should further prioritize the strategies for mitigating these structural and process QoHC delivery elements if they seek to improve MCH services. Likewise, Yevoo et al. highlight the consequences of structural and process QoHC dimensions for all types of services. The findings from this study, done at referral facilities among healthcare workers (HCWs) in Accra, Ghana, document reports from clients condemning the unethical practice of HCWs who habitually turn away patients who are brought to facilities with the expectation of being admitted for emergency care. In their remarks, the authors appeal for the responsible policy and program authorities to accord the emergency care issue the priority it deserves and to reverse the perceived negative attitude HCWs show towards the clients approaching them.

The study from Kenya and Tanzania by Mkumbo et al. highlights the implications of the absence of a universal definition of the term “*critical illness*.” They argue that this leads to variations in frontline HCWs' interpretation of the term, consequently hampering communication and the selection of patients who deserve urgent life-saving care. They justify the need for the respective HCWs to be oriented to the right definition of “critical illness” as recently highlighted by other scholars. In a similar manner, Keleb et al. report findings from a study done in public hospitals in one of the zones in Ethiopia, assessing the degree to which HCWs comply with the available standard precautions for infection prevention (IP) practice. With only about a fifth of the respondents complying, the authors suggest that more efforts need to be made to overcome the challenges faced by HCWs, which discourage them from following the recommended IP measures properly. Among the challenges noted were pervasive unreliable water supplies at the workplace and inadequate working experience.

Chekol et al. underscore the need for properly designed and optimally functioning routine health information systems (RHISs). They state that this facilitates knowledge acquisition or transferability as long as other supporting health communication channels or mechanisms are available. According to these authors, the low quality of a given RHIS-based data at the national level, compounded by the chronic complexities in the formal RHIS, such as the scarcity of key staff, results in improper routine data management and thereby compromises the utilization of data for decision making. The authors also urge the RHISs to improve feedback mechanisms in order to enhance routine data production and utilization for the betterment of the QoHC as desired.

The article by Chaker Masmoudi et al. reports findings obtained from a study done in Tunisia, focusing on the role a “multi-criteria decision analysis” (MCDA) approach. They argue that MCDA can help policy decision-makers and policy actors to identify individuals unquestionably deserving of a “most-at-risk” categorization so that they can be prioritized to receive vaccines and associated services during epidemics like COVID-19. However, these authors warn that special prioritization in relation to a vaccine or a vaccination shortage should not overlook the need to adhere to the guidelines for proper service delivery. Ultimately, data-driven MCDA can contribute significantly towards the achievement of vaccination goals with more public benefits.
